# Isolated gastric varices secondary to abdominal tuberculosis mimicking lymphoma: a case report

**DOI:** 10.1186/s12876-019-0998-9

**Published:** 2019-05-28

**Authors:** Yaoyao Gong, Shuo Li, Rong Rong, Xiaoxing Chen, Liuqin Jiang

**Affiliations:** 1 Department of Gastroenterology, The First Affiliated Hospital of Nanjing Medical University, No.300 of Guangzhou Road, Nanjing, 210029 China; 2 Department of Pathology, The First Affiliated Hospital of Nanjing Medical University, No.300 of Guangzhou Road, Nanjing, 210029 China

**Keywords:** Isolated gastric varices, Abdominal tuberculosis, Abdominal mass

## Abstract

**Background:**

Abdominal tuberculosis (TB) rarely presents with abdominal masses and rarely causes isolated gastric varices.

**Case presentation:**

We report a case of isolated gastric varices secondary to abdominal TB mimicking lymphoma. A 42-year-old woman without any history of liver disease presented with melena and mild abdominal pain. Upon admission to the hospital, laboratory investigations revealed a hemoglobin level of 76 g/L. Gastroduodenoscopic examination showed isolated gastric fundal varices with red color signs. Abdominal contrast-enhanced computed tomography (CECT) revealed non-enhanced masses of soft-tissue density in the lesser omental and the retropancreatic areas, multiple para-aortic lymph nodes, and multiple small hypodense splenic lesions. Positron emission tomography-CT showed hypermetabolic [F-18]2-fluoro-2-deoxyglucose activity involving multiple regional lymph nodes and the bone marrow, suggestive of lymphoma. Bone marrow biopsy revealed no abnormality. Histopathological examination of a CT-guided biopsy specimen showed granulomatous inflammation with necrosis and microorganisms that stained positive with acid-fast stains. Abdominal CECT showed a decrease in the size of the lesser omental and peripancreatic masses, as well as the para-aortic lymph nodes after 4-month anti-TB therapy.

**Conclusions:**

TB should be considered among the differential diagnoses in patients with abdominal masses, isolated gastric varices, and regional lymphadenopathy. Prompt and definitive diagnosis of abdominal TB requires a coordinated approach involving laboratory tests, radiological examination, and invasive procedures for optimal decision making and management.

## Background

Bleeding from isolated gastric varices is a serious condition and is usually caused by hepatic cirrhosis. Left-sided noncirrhotic portal hypertension secondary to the obstruction of the splenic vein can also precipitate isolated gastric varices. Abdominal tuberculosis (TB) is a rare form of TB that constitutes approximately 3% of the total cases of TB [1, 2]. Abdominal TB rarely presents with abdominal masses and rarely causes isolated gastric varices. We report a case of abdominal TB in a patient presenting with abdominal masses, regional lymphadenopathy, and compression of the splenic vein causing left-sided portal hypertension and the consequent development of isolated gastric varices.

## Case presentation

A 42-year-old woman was admitted to our hospital with a 1-month history of melena and mild abdominal pain. She denied nausea, vomiting, hematemesis, jaundice, cough, hemoptysis, fever, and/or menstrual abnormalities. She had no significant medical history or family history of pulmonary TB. She also denied tobacco or alcohol abuse. Upon admission, her general examination showed pallor, and abdominal examination revealed mild upper abdominal tenderness. Laboratory findings were as follows: hemoglobin 76 g/L, hematocrit 25.6%, mean corpuscular hemoglobin 25.3 pg/cell, erythrocyte sedimentation rate 36 mm/hour, and a C-reactive protein level of 24 mg/L. A T-SPOT.TB assay was performed and showed a positive result. Hepatic enzymes such as alkaline phosphatase, as well as bilirubin, albumin, prothrombin time, and partial prothrombin time showed no abnormalities. Additionally, the human immunodeficiency virus, a hepatitis panel, and tumor markers were negative. After treatment with pharmacological agents, such as proton-pump inhibitors and octreotide, active gastric variceal bleeding stopped.

**Fig. 1:**
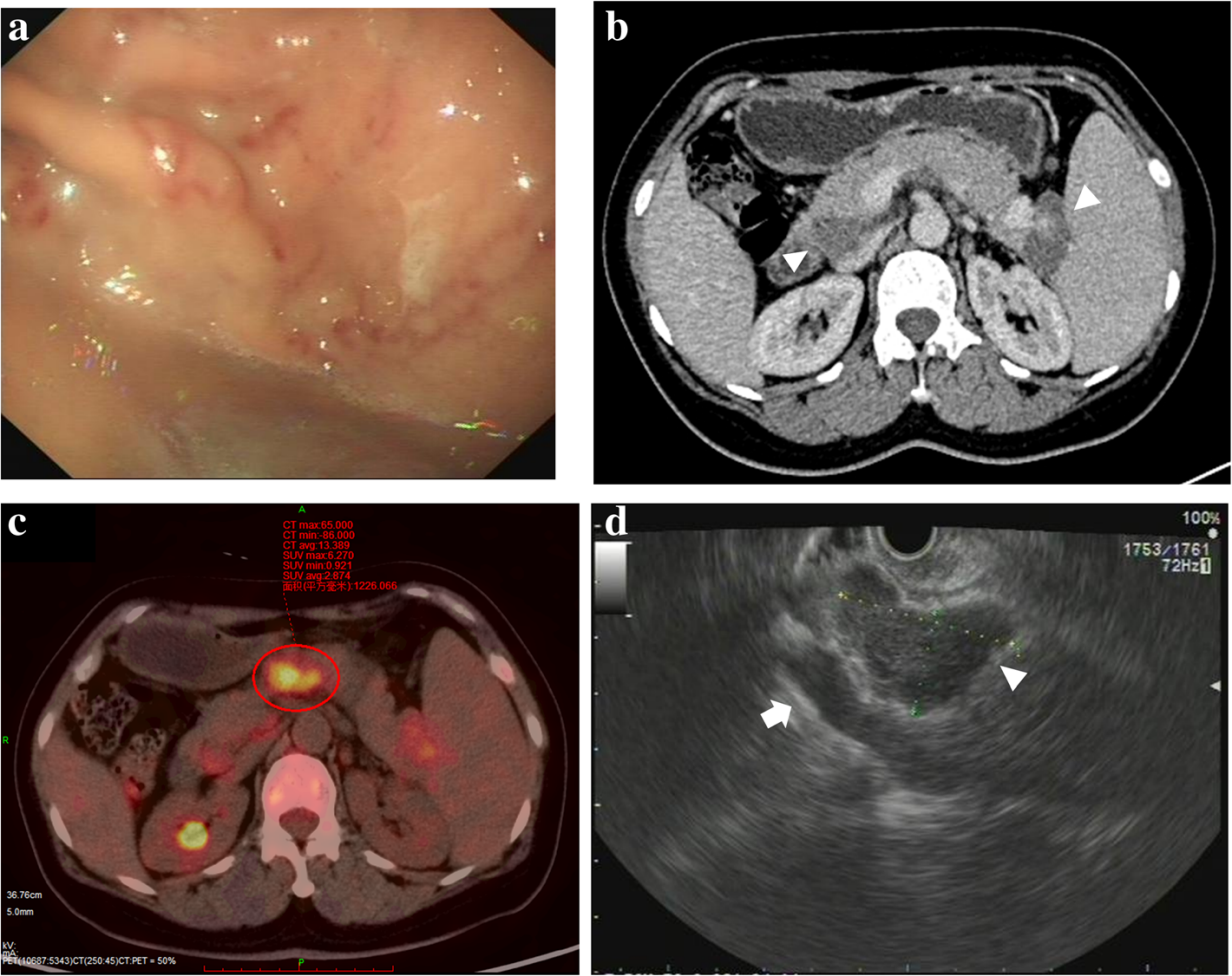
(**a**) Gastroduodenoscopic images show gastric fundal varices with characteristic red color signs. (**b**) CECT scan shows masses behind the head, body, and the tail of the pancreas (*arrow heads*). (**c**) PET/CT scan shows hypermetabolic lymphadenopathy in the retropancreatic area and at the splenic hilum (size 42.8 mm × 21.2 mm). (**d**) EUS shows a hypoechoic mass at the splenic hilum (*arrow head*) compressing the splenic vein (*arrow*)

Gastroduodenoscopic examination showed isolated gastric fundal varices with red color signs (Fig. 1a). Colonoscopic examination did not reveal any abnormality. Abdominal contrast-enhanced computed tomography (CECT) revealed non-enhanced masses of soft-tissue density in the lesser omentum and behind the head, body, and the tail of the pancreas, as well as multiple para-aortic lymph nodes and multiple small hypodense splenic lesions (Fig. 1b). CECT findings were suggestive of an infectious or metastatic disease or lymphatic cysts. Whole body [F-18]2-fluoro-2-deoxyglucose (FDG) positron emission tomography (PET)-CT was performed to evaluate for lymphoma and metastatic disease. This test showed hypermetabolic lymph nodes in the right supraclavicular fossa, right tracheoesophageal groove, the lesser omental and retropancreatic areas, the mesentery, the porta hepatis, the splenic hilum, and the retroperitoneum with maximum standardized uptake values (SUVmax) ranging from 5.3–7.5 (Fig. 1c). Additionally, the bone marrow showed hypermetabolic FDG activity with a SUVmax of 5.2. The PET/CT findings were suggestive of lymphoma.

**Fig. 2:**
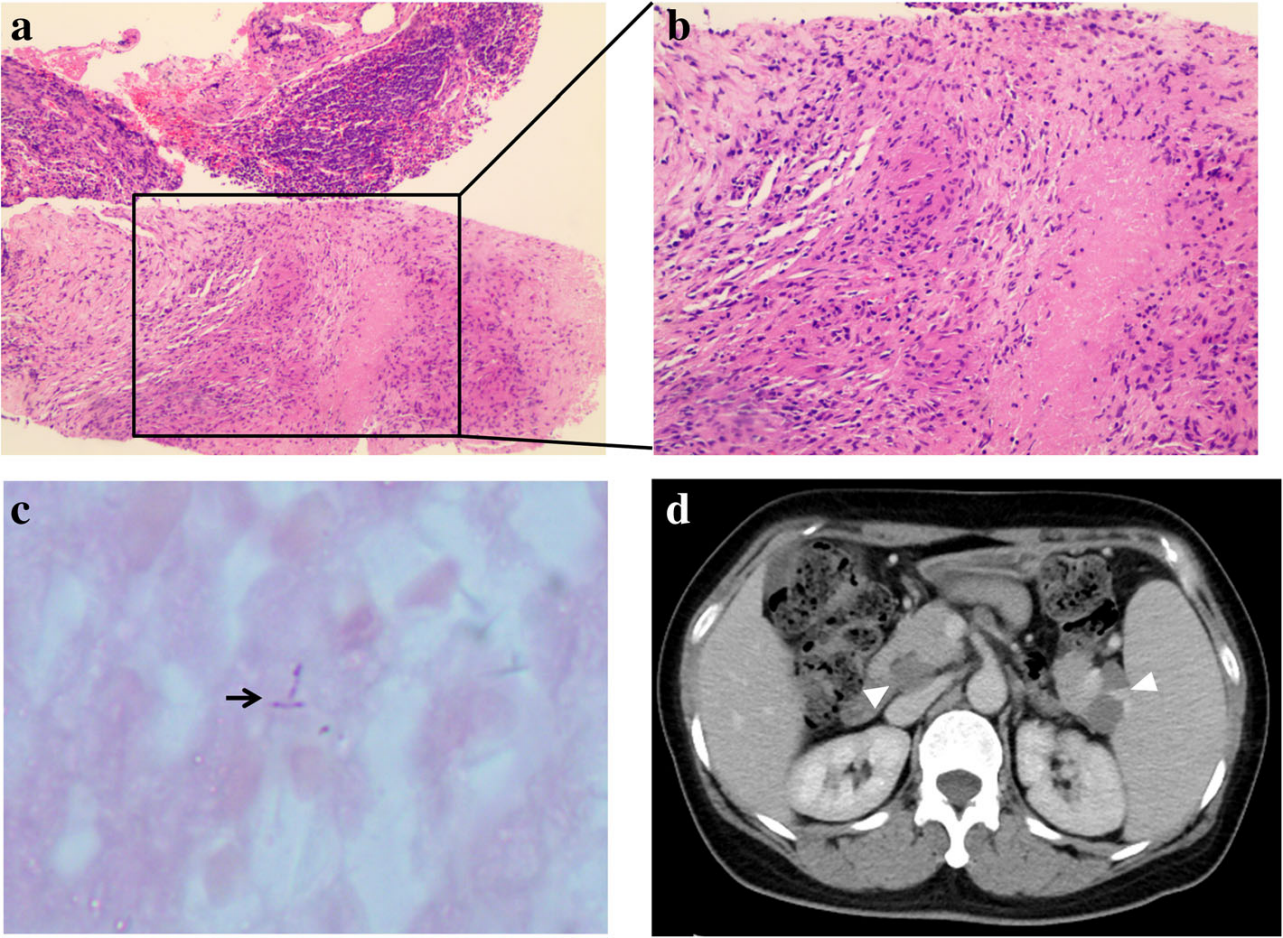
(**a**-**b**) Images of a histopathological specimen show granulomatous inflammation and granuloma formation (HE stain [× 100, × 200]). (**c**) Microorganisms positively stained with acid-fast stains can be observed (arrow) (× 1000). (**d**) CECT scan shows a decrease in the size of the masses in the retropancreatic area and at the splenic hilum (size 37.0 mm × 16.0 mm) after 4-month anti-TB therapy

Bone marrow biopsy revealed no abnormality. Subsequently, endoscopic ultrasonography (EUS) was performed and revealed a hypoechoic mass at the splenic hilum, compressing the splenic vein (Fig. 1d). EUS-guided fine-needle aspiration was performed to obtain tissues for histopathological examination. Histopathological examination showed sheets of inflammatory cells, primarily lymphocytes and macrophages along with some glandular tissue. CT-guided biopsy of the retroperitoneal lymph nodes was performed to establish a definitive diagnosis, and the histopathological examination of these biopsy specimens showed necrotizing granulomatous inflammation and microorganisms that stained positive with acid-fast stains (Fig. 2a-c).

Based on all the aforementioned findings, she was diagnosed with abdominal TB. Anti-TB treatment comprising rifampicin, isoniazid, and ethambutol was initiated following histopathological confirmation. After leaving our hospital, she went to a TB hospital for further treatment, where her doctor added pyrazinamide to the treatment. She received a 6-month regimen of rifampicin: 2HRZE/4HR. During the course of treatment, the patient experienced mild elevation of transaminase. Abdominal CECT showed a decrease in the size of the lesser omental and retropancreatic masses, as well as the para-aortic lymph nodes after 4-month anti-TB therapy (Fig. 2d). The patient was asymptomatic at her 12-month follow-up.

## Discussion and conclusions

TB remains a global health issue particularly in developing countries. Abdominal TB is a relatively rare manifestation of TB. Patients with abdominal TB commonly present with abdominal pain and distention, fever, ascites, weight loss, fatigue, anorexia, diarrhea, and anemia [2]; however, they rarely present with gastric fundal varices. This patient presented with sudden-onset melena accompanied by mild abdominal pain, but without any typical TB symptoms. The cause of melena was gastric fundal varices, which were identified by gastroduodenoscopy. Abdominal TB involving the portal vasculature is a rare phenomenon. In the present case, EUS showed a hypoechoic mass in the splenic hilum compressing the splenic vein as the cause of portal hypertension that subsequently led to gastric fundal varices. Moreover, no specific risk factors for TB were identified in this patient.

Previous studies have reported that the porta hepatis, peripancreatic, and para-aortic regions are the most common abdominal sites affected by TB lymphadenitis [3]. In this patient, PET/CT findings showed that lymph nodes in the supraclavicular fossa and the tracheoesophageal groove were involved in addition to typical abdominal lymph node involvement. Moreover, the rate of bone marrow FDG uptake was increased along with the increased FDG uptake in lymph nodes. Notably, abdominal TB rarely presents with abdominal masses. In this case, abdominal CECT showed lesser omental and retropancreatic masses. It should be noted that TB lymphadenitis usually shows low-attenuation masses with a rim of peripheral enhancement on CECT [4]; however, the lesser omental and retropancreatic masses did not show the typical peripheral enhancement in our patient. The PET/CT findings suggested malignant disease, particularly lymphoma. PET/CT scanning is useful for distinguishing malignant lesions in the brain, head and neck, breast, and liver from other types of lesions. In this case, PET/CT findings were not useful for distinguishing tuberculous lymphadenitis from lymphoma. Based on the present case, even if imaging findings suggest lymphoma, histopathological examination and acid-fast staining still may be useful to exclude TB.

After diagnosis of extrapulmonary TB, a 6-month course of anti-TB therapy is recommended according to WHO TB treatment guidelines [5]. Abdominal TB usually has a high cure rate after 6- or 9-month standard anti-TB therapy [1, 6]. However, in some studies, abdominal TB has a high mortality rate (9–29.8%) [7–9]. As reported by previous studies, the prognosis of abdominal TB depends on prompt diagnosis and treatment [2, 10]. We could establish the final diagnosis in our patient in 23 days, which is sooner than the period mentioned in previous reports [2]. Diagnosis of abdominal TB is difficult owing to the vague and non-specific clinical features of this disease and the low rates of *Mycobacterial* smear and culture positivity [11]. Furthermore, invasive procedures are required to obtain tissues for histopathological examination. Therefore, diagnosis of abdominal TB remains challenging. The T-SPOT.TB assay has been widely used in China in recent years as a diagnostic tool for TB infection. The T-SPOT.TB assay shows high sensitivity and scores over bacterial cultures to diagnose smear-negative TB [12]. Prompt and definitive diagnosis of abdominal TB requires a coordinated approach involving laboratory tests (notably the T-SPOT.TB assay), radiological examination, and invasive procedures for optimal decision making and management.

In conclusion, abdominal TB rarely presents with abdominal masses and causes isolated gastric varices. We report this case to serve as a useful reference for clinicians who might encounter clinically similar conditions. In addition to cancer and lymphoma, abdominal TB is a possible diagnosis for patients with abdominal masses and isolated gastric varices, even if the abdominal masses do not show peripheral rim enhancement on CECT.

## Data Availability

Data sharing is not applicable to this article as no datasets were generated or analyzed during the current study.

## Abbreviations

CECT: Contrast-enhanced computerized tomography
EUS: Endoscopic ultrasonography
FDG: [F-18]2-fluoro-2-deoxyglucose
FNA: Fine-needle aspiration
PET: Positron emission tomography
SUVmax: Maximum standardized uptake value
TB: Tuberculosis
